# Antibacterial Mechanisms and Efficacy of Sarecycline in Animal Models of Infection and Inflammation

**DOI:** 10.3390/antibiotics10040439

**Published:** 2021-04-15

**Authors:** Christopher G. Bunick, Jonette Keri, S. Ken Tanaka, Nika Furey, Giovanni Damiani, Jodi L. Johnson, Ayman Grada

**Affiliations:** 1 Department of Dermatology, Yale University School of Medicine, New Haven, CT 06510, USA; christopher.bunick@yale.edu; 2 Department of Dermatology and Cutaneous Surgery, University of Miami Miller School of Medicine, Miami, FL 33128, USA; JKeri@med.miami.edu; 3 Paratek Pharma, Boston, MA 02109, USA; Ken.Tanaka@paratekpharma.com; 4 R&D and Medical Affairs, Almirall US, Exton, PA 19341, USA; nika.furey@almirall.com; 5 Clinical Dermatology, IRCCS Galeazzi Orthopaedic Institute, Via Riccardo Galeazzi, 4, 20161 Milan, Italy; dr.giovanni.damiani@gmail.com; 6 Department of Biomedical, Surgical and Dental Sciences, University of Milan, 20121 Milan, Italy; 7 Department of Pharmaceutical and Pharmacological Sciences, University of Padua, 35139 Padua, Italy; 8 Departments of Pathology & Dermatology, Feinberg School of Medicine, Northwestern University, Chicago, IL 60602, USA; jodi-johnson@northwestern.edu

**Keywords:** antibiotic, sarecycline, tetracyclines, narrow-spectrum, infection, inflammation, antibiotic resistance, acne, animal models

## Abstract

Prolonged broad-spectrum antibiotic use is more likely to induce bacterial resistance and dysbiosis of skin and gut microflora. First and second-generation tetracycline-class antibiotics have similar broad-spectrum antibacterial activity. Targeted tetracycline-class antibiotics are needed to limit antimicrobial resistance and improve patient outcomes. Sarecycline is a narrow-spectrum, third-generation tetracycline-class antibiotic Food and Drug Administration (FDA)-approved for treating moderate-to-severe acne. In vitro studies demonstrated activity against clinically relevant Gram-positive bacteria but reduced activity against Gram-negative bacteria. Recent studies have provided insight into how the structure of sarecycline, with a unique C7 moiety, interacts with bacterial ribosomes to block translation and prevent antibiotic resistance. Sarecycline reduces *Staphylococcus aureus* DNA and protein synthesis with limited effects on RNA, lipid, and bacterial wall synthesis. In agreement with in vitro data, sarecycline demonstrated narrower-spectrum in vivo activity in murine models of infection, exhibiting activity against *S. aureus*, but reduced efficacy against *Escherichia coli* compared to doxycycline and minocycline. In a murine neutropenic thigh wound infection model, sarecycline was as effective as doxycycline against *S. aureus*. The anti-inflammatory activity of sarecycline was comparable to doxycycline and minocycline in a rat paw edema model. Here, we review the antibacterial mechanisms of sarecycline and report results of in vivo studies of infection and inflammation.

## 1. Introduction

Tetracycline-class antibiotics, such as tetracycline, doxycycline, and minocycline, have been commonly prescribed to treat both Gram-positive and Gram-negative bacterial infections [1]. Broad-spectrum antibiotics select for antimicrobial resistance in a larger, more diverse microbiome compared to a narrow-spectrum antibiotic. Broad-spectrum antibiotics have been associated with microbial dysbiosis and put patients at risk of antibiotic-associated adverse events (AEs) [2,3,4]. The reported modes of bacterial resistance to tetracycline-class antibiotics include efflux (export by efflux pumps), ribosomal protection proteins (catalyze Guanosine-5’-triphosphate (GTP)-dependent release of ribosome-bound tetracycline allowing translation to proceed), ribosomal mutations (can reduce connection between tetracycline and the ribosome), and tetracycline inactivating enzymes (covalently modify the tetracycline scaffold rendering tetracycline inactive) [5]. Tetracycline-resistant bacteria are rapidly increasing [6,7,8,9]. Dermatologists prescribe more oral antibiotic courses per clinician than any other specialty, with tetracycline agents accounting for around 75% of all antibiotic prescriptions written by dermatologists [3].

Moderate-to-severe acne vulgaris is a chronic inflammatory disease commonly treated with oral tetracycline-class antibiotics, particularly in cases of lesions arising on the back and chest [1,10]. Most antibiotics from the tetracycline family share similar chemical structures and mechanisms of action (MOA). These broad-spectrum antibiotics demonstrate nonspecific activity, inhibiting bacteria that comprise the healthy gut microflora. This can cause dysbiosis and, hence, gastrointestinal side effects, such as diarrhea, nausea, vomiting, and potentially chronic diseases, such as inflammatory bowel disease [11,12,13,14,15,16]. Prolonged use of broad-spectrum tetracyclines may also cause vulvovaginal mycotic infections and candidiasis [17,18]. In addition, doxycycline and minocycline can cross the blood–brain barrier, causing vestibular side effects, such as headaches, dizziness, and pseudotumor cerebri [19,20,21]. The AEs and potential for bacterial resistance caused by most tetracycline-class antibiotics highlight a distinct unmet need for novel targeted antibiotics to treat acne vulgaris and provide safer and effective alternatives to enhance patient outcomes [22].

Sarecycline is a narrow-spectrum tetracycline-based antibiotic with an enhanced MOA due to its unique chemical structure. Sarecycline was approved specifically for treatment of acne vulgaris in 2018, becoming the first new Food and Drug Administration (FDA)-approved antibiotic for acne in almost half a century [23,24]. The safety, efficacy, dosage, pharmacokinetics, and pharmacodynamics have been reviewed and published elsewhere [25]. Here we review the data for sarecycline structure and MOA, as well as the in vivo efficacy of sarecycline against *Staphylococcus aureus (S. aureus)* and *Escherichia coli* (*E. coli*) in animal models of infection. We also reveal the anti-inflammatory activity of sarecycline in a rat footpad edema model. Together, these studies demonstrate that sarecycline preferentially inhibits Gram-positive *S. aureus* to effectively reduce infection and tissue inflammation in animal models.

## 2. Sarecycline Structure and Mechanism of Action

**Figure 1:**
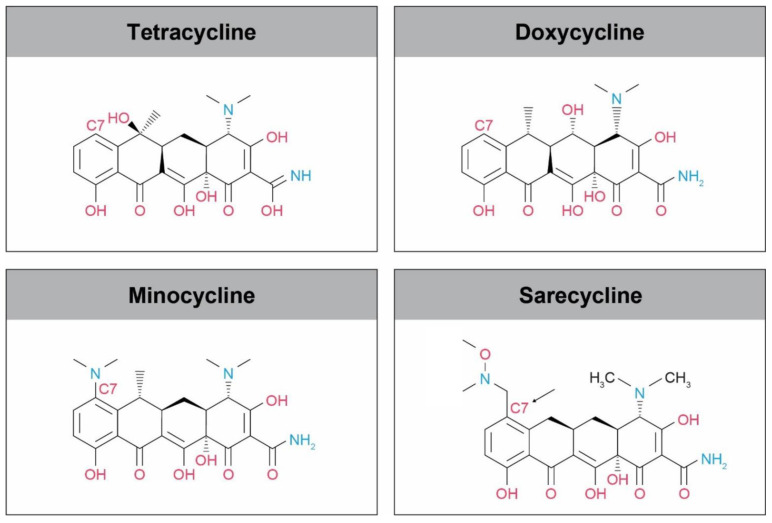
Chemical structures of the different tetracycline-class drugs used for the treatment of acne vulgaris: tetracycline, doxycycline, minocycline, and sarecycline. The incorporation of a longer C7 moiety allows sarecycline to interact more strongly with the bacterial ribosome. Structure image source: http://www.chemspider.com/Chemical-Structure.28540486.html (accessed on 14 April 2021).

Tetracycline-class drugs share a common core structure consisting of four hydrocarbon rings. Tetracyclines can be distinguished from one another by different types of functional groups attached to the hydrocarbon rings, which specify how each drug contributes to antibacterial activity (Figure 1) [26,27]. Sarecycline hydrochloride is a new chemical entity with the chemical name of (4S,4aS,5aR,12aS)-4-(Dimethylamino)-3,10,12,12a-tetrahydroxy-7-[(methoxy-(methyl)-amino)-methyl]-1,11-dioxo-1,4,4a,5,5a,6,11,12a-octahydrotetracene-2-carboxamide monohydrochloride (C_24_H_29_N_3_O_8_·HCl). The drug is produced as the hydrochloride (HCl) salt. Unlike other tetracyclines, the chemical structure of sarecycline includes a stable modification, a 7-[(methoxy-(methyl)-amino)-methyl]methyl] group at hydrocarbon ring C7 (Figure 1). To date, sarecycline has the longest and largest C7 moiety present in any tetracycline-class drug [26,28].

**Figure 2:**
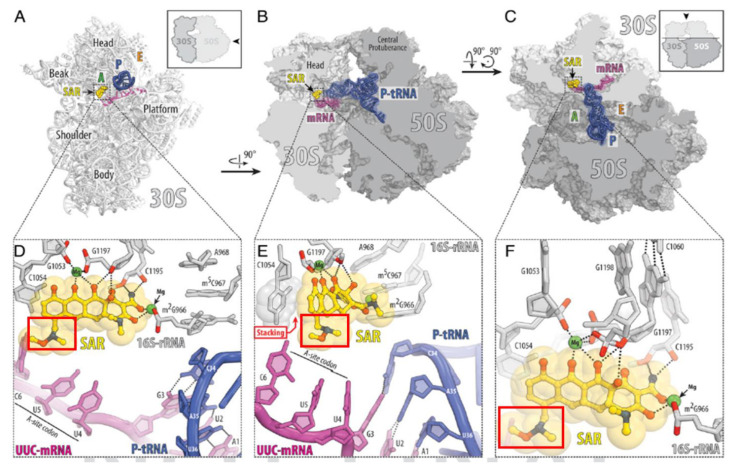
Atomic resolution crystallographic structure of sarecycline in complex with the bacterial 70S ribosome. (**A**–**C**) Overview of the SAR binding site (yellow) on the T. thermophilus 70S ribosome viewed from three different perspectives. The 30S subunit is shown in light gray, the 50S subunit is dark gray, the mRNA is magenta, and the P site tRNA is colored dark blue. In A, the 30S subunit is viewed from the intersubunit interface, as indicated by Inset (the 50S subunit and parts of the P site tRNA are removed for clarity). The view in B is a transverse section of the 70S ribosome. The view in C is from the top after removing the head of the 30S subunit and protuberances of the 50S subunit, as indicated by Inset. Red boxes in (**D**–**F**) indicate the C7 moiety of sarecycline. mRNA is shown in purple. Image used with permission from [26].

Tetracycline-class antibiotics can inhibit bacterial protein synthesis by interacting with the 30S subunit of the 70S bacterial ribosome using their functional groups, but sarecycline’s modified C7 moiety gives it an enhanced mode of action. A recent X-ray crystallography study [26] demonstrated that unlike other tetracyclines, sarecycline’s C7 moiety extends into the mRNA channel within the ribosome and forms a direct interaction with the A site codon, possibly interfering with mRNA movement through the channel and/or disrupting A site codon-anticodon interaction (Figure 2). Sarecycline exhibits increased stabilization on the bacterial 70S ribosome, thereby blocking tRNA accommodation and potently inhibiting mRNA translation [26,28].

## 3. In vitro Inhibition of Bacterial Biosynthetic Endpoints

Tetracycline-class antibiotics exhibit bacteriostatic activity primarily by targeting protein synthesis to inhibit bacterial growth and activity. The tetracyclines, including sarecycline, bind the 16S rRNA of the small 30S ribosomal subunit to inhibit initiation of translation [26,29]. Tetracyclines inhibit bacterial protein synthesis by inhibiting the binding of aminoacyl-tRNA to the mRNA-ribosome complex. Sarecycline inhibits protein synthesis by causing most ribosomes to stall at the initiation codon, with a small fraction of ribosomes escaping and continuing with elongation [26,28]. Sarecycline may exhibit higher binding affinity to ribosomes than other tetracyclines, making it a more potent translational inhibitor [26].

**Figure 3:**
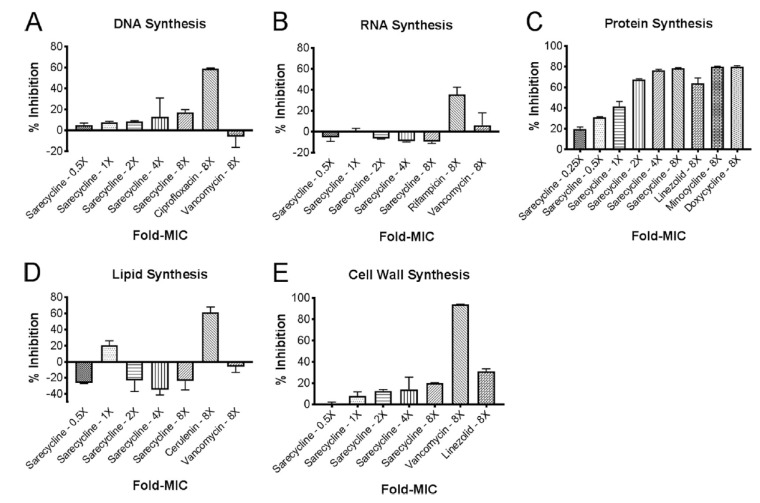
Effect of sarecycline on macromolecular biosynthesis in (**A**) DNA synthesis, (**B**) RNA synthesis, (**C**) protein synthesis, (**D**) lipid synthesis, and (**E**) cell wall synthesis in *S. aureus* ATCC 29213. DNA, RNA, protein, cell wall, and lipid synthesis was determined by measuring incorporation of (3H)thymidine, (3H)uridine, (3H)leucine, (3H)*N*acetylglucosamine, and (3H)glycerol, respectively. Control agents included ciprofloxacin (a DNA synthesis inhibitor), linezolid (a protein synthesis inhibitor), cerulenin (a lipid synthesis inhibitor), vancomycin (a cell wall biosynthesis inhibitor), and rifampin (a RNA synthesis inhibitor). Data represent the median with 95% confidence intervals (*n* = 3). Image used with permission from [30].

Sarecycline has been tested for its ability to inhibit major biosynthetic endpoints, including DNA, RNA, and protein synthesis as well as lipid and bacterial wall synthesis (Figure 3) [30]. In *S. aureus,* DNA synthesis was inhibited by 20% at 8-fold the minimal inhibitory concentration (MIC) of sarecycline, though ciprofloxacin (used as a positive control) inhibited DNA synthesis by 80% (Figure 3A). Thus, in addition to inhibiting translation, sarecycline has a low-level ability to inhibit transcription as well. In previous studies, tetracycline inhibited DNA synthesis in *E. coli* and *Bacillus subtilis* at concentrations much higher than required for inhibiting protein synthesis [31]. Sarecycline showed limited effects on RNA synthesis, lipid biosynthesis, and cell wall biosynthesis in *S. aureus* (Figure 3B,D,E). However, sarecycline inhibited protein synthesis in *S. aureus* in a dose-dependent manner that reached a maximum inhibition of 80% at 8-fold of the MIC, similar to minocycline and doxycycline (Figure 3C).

Sarecycline inhibited prokaryotic transcription-coupled protein translation in vitro as measured using a Transcription/Translation (TnT) assay. The TnT assay makes use of an *E. coli* S30 Extract system (Promega Corporation, Madison, WI) and a modified circular plasmid template DNA (pBEST luc) containing the firefly luciferase gene under control of the *E. coli* promoter. Translation is monitored by luciferase expression quantitatively measured using a luminescence microplate reader (Wallac). Briefly, sarecycline, doxycycline, and minocycline were dissolved to 20 mM in DMSO and diluted to 1 mM, 0.5 mM, 0.25 mM, 0.1 mM, 0.01 mM, and 0.001 mM. Diluted compounds (5 µL) were used in a 50 µL reaction to yield final concentrations of 100 µM, 50 µM, 25 µM, 10 µM, 1 µM, and 0.1 µM, respectively. Following incubation with 0.5 µg of pBEST luc DNA, complete amino acid mixture, and *E. coli* S30 for 25 min at 37 °C, 50 µL of luciferase dilution reagent was added. A total of 15 µL of the mixture was assayed with 50 µL Luciferase Assay Reagent and luciferase expression was read on a luminescence microplate reader within 10 min. All samples were assayed in triplicate, with data presented as mean ± standard error. The half maximal inhibitory concentration (IC_50_) values of compounds were determined by XcelFit, representing the concentration at which 50% inhibition is observed relative to non-drug treated controls. In *E. coli*, protein synthesis was inhibited by sarecycline at an IC_50_ of 8.3 ± 0.18 µM, which was comparable to, but slightly higher than the IC_50_ values for doxycycline and minocycline (4.7 ± 0.48 and 2.4 ± 0.22 µM, respectively). These data have not previously been published and are on file with Almirall.

Taken together, these data indicate that sarecycline retains the classical ability of tetracyclines to primarily inhibit bacterial protein translation to cause its antibacterial effect and has some ability to inhibit DNA synthesis.

## 4. In vitro Antibacterial Effects

**Figure 4:**
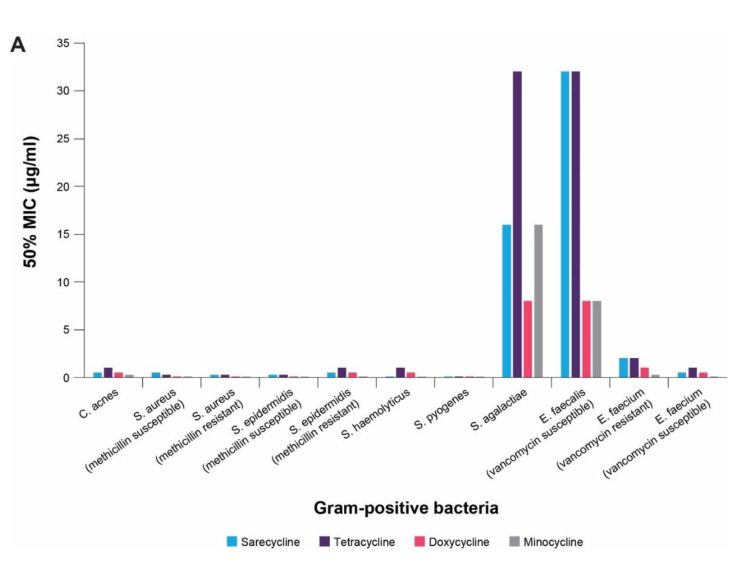

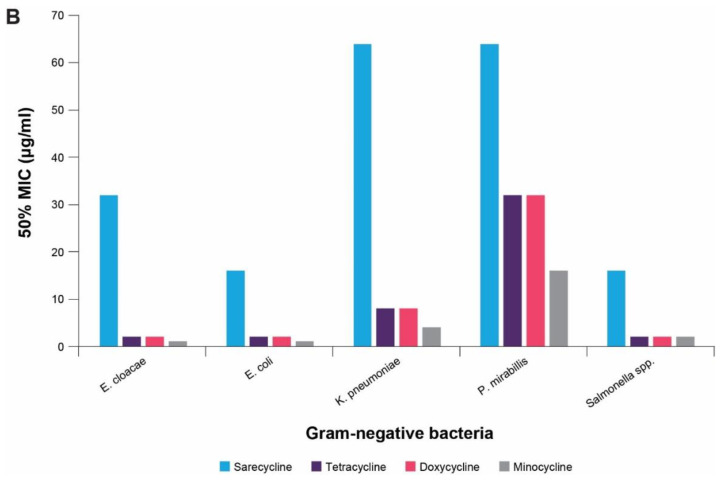
Minimum inhibitory concentration required to inhibit the growth of 50% of organisms (MIC_50_) of sarecycline and other tetracycline-class antibiotics in (**A**) Gram-positive bacteria and (**B**) Gram-negative bacteria. The lower the MIC_50_, the more effective the antibiotic against that bacterial type. Data originally reported in [30].

Sarecycline has demonstrated targeted activity against Gram-positive bacteria, including *Cutibacterium acnes* (*C. acnes*), the key bacteria associated with acne pathogenesis [30]. In vitro, sarecycline activity against *C. acnes* clinical isolates had a comparable minimum inhibitory concentration required to inhibit the growth of 50% of organisms (MIC_50_) (0.5 µg/mL) to other tetracyclines, including minocycline (0.25 µg/mL), doxycycline (0.5 µg/mL), and tetracycline (1 µg/mL) (Figure 4A). MIC_50_ was considered to represent the intrinsic activity of each antimicrobial. Sarecycline also demonstrated similar activity against clinical isolates of *S. aureus* including methicillin-resistant strains (MRSA) (Figure 4A), which can colonize damaged tissue in acne patients [32,33]. In contrast to broad-spectrum tetracyclines, sarecycline has shown reduced activity against enteric bacteria, resulting in 16 to 32-fold less activity against aerobic Gram-negative bacteria when compared to doxycycline and minocycline (Figure 4B) [30]. The difference in activity against Gram-positive and Gram-negative bacteria could be attributed to the intrinsic difference in the permeability of the outer bacterial wall membrane with sarecycline being less permeable to the outer wall of Gram-negative bacteria [5]. Sarecycline was much less effective against aerobic Gram-negative bacteria than other tetracyclines. Against *E. coli,* the MIC_50_ of sarecycline was 16 µg/mL, while the MIC_50_ was between 1 and 2 µg/mL in the other tetracyclines. Additionally, sarecycline has 4 to 8-fold less activity against anaerobic bacteria comprising the human intestinal microflora, including *Bifidobacterium bifidum*, *Clostridium difficile*, and *Lactobacillus acidophilus.* This may result in decreased gut dysbiosis [26,30]. Indeed, in the pivotal Phase 3 clinical trials as well as the open-label long-term 40 weeks extension safety study, sarecycline showed a low rate of AEs commonly associated with broad-spectrum tetracycline-class antibiotics, including low rates of gastrointestinal upset [16,34,35].

## 5. In vivo Antibacterial Efficacy

Since no accepted animal model of acne has been developed to date, the efficacy of sarecycline in vivo was assessed using infection models in CD-1 mice [30]. Sarecycline demonstrated efficacy against Gram-positive organisms comparable to or slightly better than doxycycline in murine infection models. Efficacy in these models—determined by PD_50_ (protective dose required to achieve 50% survival) or ED_50_ (effective dose required to achieve a 50%, or 2-log_10_, reduction in bacterial burden)—was observed. A murine systemic (intraperitoneal) infection model was used to assess the in vivo efficacies of sarecycline, doxycycline, and minocycline against *S. aureus* and *E. coli*. At 48 h after systemic infection with *S. aureus,* sarecycline, doxycycline, and minocycline had a PD_50_ of 0.25, 0.3, and 0.03 mg/kg, respectively (Table 1) [30]. In contrast, sarecycline did not demonstrate in vivo efficacy against *E. coli* even at the highest dose (PD_50_ > 40 mg/kg), while doxycycline and minocycline had a PD_50_ of 5.72 and 6.95 mg/kg, respectively (Table 1) [30]. Furthermore, a murine neutropenic thigh wound infection model was utilized as a tissue-based infection to assess efficacies of sarecycline and doxycycline against *S. aureus*. At 24 h post infection, sarecycline achieved a 2-log_10_ reduction in thigh bacterial burden comparable to doxycycline, with 50% effective dose (ED_50_) values of 8.23 and 8.32 mg/kg, respectively (Table 2) [30]. These in vivo results highlight a narrower-spectrum of activity of sarecycline when compared to doxycycline and minocycline, in agreement with the in vitro results.

## 6. Antimicrobial Resistance Profile

The four main mechanisms through which bacteria become resistant to tetracyclines are through efflux, degradation, rRNA mutations, and ribosomal protection [36,37]. Efflux pumps present in both Gram-negative and Gram-positive bacteria, including the well characterized tetracycline efflux pump TetA, pump tetracyclines out of the bacterial cell. Monooxygenases, present in bacteria growing in aerobic conditions, can hydroxylate tetracyclines, reducing the binding affinity of the drug to the ribosome. rRNA mutations (within the 16S rRNA) can reduce the binding affinity of tetracyclines to the ribosome. Lastly, ribosomal protection can occur via ribosomal protection proteins (RPPs), including the well characterized TetO and TetM, which disrupt tetracycline binding. TetO and TetM confer resistance by dislodging tetracycline from the 70S ribosome [38,39]. TetM dislodges tetracycline from its binding pocket through an overlap of its Tyr506-Ser508-Pro509-Val210 residue(s) at the tip of loop 3 in domain IV with the binding position of tetracyclines.

Recent evidence suggests that the structural modifications of sarecycline can overcome antibiotic resistance through activity against RPPs. The C7 extension of sarecycline clashes with TetM residues, inhibiting their activity. Sarecycline potentially exhibits higher affinity to the 70S ribosome compared to other tetracyclines based on toe-printing experiments [26].

In vitro, sarecycline has been shown to be more active compared with other tetracyclines against efflux [30]. Acquisition of the gene *tet*(K), *tet*(L), or *tet*(38) confers the ability for active efflux and *tet*(M), *tet*(O), *tet*(S), or *tet*(W) for ribosomal protection. In *S. aureus*, a combination of *tet*(M) and *tet*(K) most commonly confers tetracycline resistance. In strains of *S. aureus* commonly resistant to tetracyclines, sarecycline was more active than tetracycline against *tet*(K) strains, with the MICs ranging between 0.12 and 0.5 µg/mL and those of tetracycline ranging between 16 and 65 µg/mL. Sarecycline also displayed elevated MICs against strains containing a combination of *tet*(M) and *tet*(38) similar to other tetracyclines. Taken together, sarecycline may be able to combat resistance through activity hindering both efflux and ribosomal protection [26,30].

In spontaneous mutation frequency studies, *C. acnes* strains displayed a low propensity for developing resistance to sarecycline, with spontaneous mutation frequencies being 10^−10^ at 4–8 × MIC [24,30]. Sarecycline also showed a low spontaneous mutation frequency of 10^−9^ for *S. aureus* at 4- and 8-fold the MIC and 10^−8^ for *S. epidermidis* at 2- and 8-fold the MIC [30].

## 7. In-Vivo Anti-Inflammatory Effects

Tetracycline-class antibiotics are well known for their anti-inflammatory activity, which is mediated through a number of mechanisms including the inhibition of neutrophil activation and chemotaxis, inhibition of matrix metalloproteinases (MMPs), and downregulation of inflammatory cytokines (Table 3) [1,40,41]. Inhibition of MMPs is the most common anti-inflammatory property of tetracyclines; this includes inhibition of collagenases and gelatinases by doxycycline, and less potently by tetracycline and minocycline [41,42]. Tetracyclines inhibit MMPs by both direct inhibition and inhibition of synthesis, though specific mechanisms remain unknown. At relatively lower concentrations, tetracycline, doxycycline, and minocycline have been shown to inhibit bacterial production of neutrophil chemoattractants, including peptide chemotactic factor and lipase [41,43]. Tetracyclines have also been shown to inhibit leukocyte migration [41].

Suppression of T-cell proliferation, in a dose-dependent manner, has been demonstrated by minocycline [41]. In addition, minocycline has been investigated for its antioxidant properties in reactive oxygen species (ROS) scavenging [41]. At pharmacological doses, minocycline and doxycycline have been shown to inhibit granuloma formation [43]. While the mechanism of this inhibition is unknown, it may relate to inhibition of protein kinase C, which is commonly involved in inflammation. Lastly, cytokines are involved in inflammation and are inhibited by tetracyclines. Doxycycline has been shown to inhibit tumor necrosis factor α, interleukin (IL)-1β, and IL-6. Tetracycline has been shown to inhibit IL-8. A summary of anti-inflammatory mechanisms of action of tetracyclines in shown in Table 3.

In vivo, minocycline and doxycycline have been shown to decrease inflammation in the rat carrageenan-induced footpad edema model, a model commonly used to analyze the anti-inflammatory properties of experimental drugs [45,46]. To determine whether sarecycline also decreased inflammation, male Sprague Dawley rats were intraperitoneally injected with saline, sarecycline, or a positive control (either doxycycline or minocycline) followed 5 min later by a subplantar injection of sterile 1 mg/0.1 mL carrageenan solution in the right hind paw. Paw surface volume was measured immediately after carrageenan injection using a digital water plethysmometer (LE7500, PanLab/Harvard Apparatus), and again 3 h later. Rats were checked over the course of 3 h and cared for under the policies and guidelines given by the Paratek Institutional Animal Care and Use Committee (IACUC) and the Animal Facility Procedures. Rats were euthanized via CO_2_ asphyxiation followed by cervical dislocation immediately after the second measurement. Percent inflammation was calculated as 100 × ((post paw volume at 3 h—pre paw volume at 0 h)/pre paw volume at 0 h). Mean percent inflammation at a dose of 100 mg/kg was reduced to 53.1%, 36.0%, and 20.5% for sarecycline, doxycycline, and minocycline, respectively, compared to baseline. For a dose of 75 mg/kg, mean percent inflammation was reduced to 55.7%, 67.6%, and 53.9%, respectively (Table 4). Thus, in the rat paw edema model, sarecycline demonstrated anti-inflammatory activity comparable to doxycycline and minocycline at all doses tested. These results have not previously been published and are on file with Almirall. Sarecycline has also demonstrated efficacy in reducing inflammatory acne lesions in pivotal clinical trials [47].

## 8. Conclusions

Sarecycline is a novel, narrow-spectrum tetracycline-based antibiotic that is FDA approved for the treatment of acne vulgaris. Sarecycline has activity against clinically relevant Gram-positive bacteria but reduced activity against Gram-negative bacteria commonly found in the human gut. Recent work revealed that the unique C7 moiety of sarecycline contributes to its antibacterial activity particularly through its increased affinity for ribosome binding and interference with mRNA’s ability to move through the ribosomal channel. Sarecycline structural properties may help overcome common tetracycline resistance mechanisms. In vivo studies using animal models of infection confirmed sarecycline’s narrow spectrum of activity previously shown in vitro. Importantly, sarecycline showed anti-inflammatory activity comparable to doxycycline and minocycline in a rat model of inflammation. Altogether, these data illustrate the great potential of sarecycline for treatment of acne vulgaris with a targeted spectrum of activity, lower risk of antibiotic resistance, and fewer AEs than other tetracycline-class antibiotics. Narrow-spectrum antibiotics offer an alternative for clinicians to improve antibiotic stewardship and limit bacterial resistance.

## Figures and Tables

**Table 1:** Efficacy of sarecycline and other tetracycline-class antibiotics against *S. aureus* and *E. coli* in a murine systemic model of infection at 48 h post-infection.

Antibacterial Agent	*S. aureus* RN450-1		*E. coli* PBS1478	
	MIC (μg/mL)	PD_50_ (mg/kg)	MIC (μg/mL)	PD_50_ (mg/kg)
Sarecycline	≤0.06	0.25	4	>40
Doxycycline	≤0.06	0.3	0.5	5.72
Minocycline	≤0.06	0.03	1	6.95

Sarecycline proved effective against *S. aureus* (Gram-positive bacteria) in a murine systemic infection model. However, low efficacy of sarecycline was demonstrated vs. *E. coli* (Gram-negative enteric bacteria). The comparator drugs were effective against *E. coli* at doses ≤7 mg/kg. MIC—minimum inhibitory concentration; PD50—protective dose required to achieve 50% survival. Table adapted from [30].

**Table 2:** Efficacy of sarecycline and doxycycline against *S. aureus* in a murine neutropenic thigh infection model.

Antibacterial Agent	MIC (μg/mL)	ED_50_ (mg/kg)
Sarecycline	≤0.06	8.23
Doxycycline	≤0.06	8.31

MIC—minimum inhibitory concentration; ED50—effective dose required to achieve a 50%, or 2-log_10_, reduction in bacterial burden. Minocycline was not tested in this experiment. Table adapted from [30].

**Table 3:** Reported anti-inflammatory effects of tetracycline-class antibiotics.

Inflammatory Mechanism of Action
Inhibition of bacterial products stimulating inflammation
Suppression of neutrophil migration and chemotaxis
Inhibition of T-lymphocyte activation and proliferation
Inhibition of phospholipase A2
Inhibition of MMP
Inhibition of mast cell activation
Reactive oxygen species scavenging
Suppression of pro-inflammatory cytokine release (TNFα, IL-1β, IL-6, IL-8)
Inhibition of granuloma formulation in vitro
Inhibition of expression of nitric oxide synthase
Inhibition of angiogenesis in mouse models

IL, interleukin; MMP, matrix metalloproteinase; TNFα, tumor necrosis factor alpha. Table adapted from [41,44].

**Table 4:** Inhibition of inflammation in a carrageenan-induced rat footpad edema model.

Compound	Mean Percent Inflammation Compared to Untreated Controls							
	150 mg/kg	100 mg/kg	75 mg/kg	50 mg/kg	25 mg/kg	10 mg/kg	5 mg/kg	1 mg/kg
Sarecycline	25.8	53.1	55.7	52.0	59.0	65.2	77.8	103.3
Doxycycline	-	36.0	67.6	-	-	-	-	-
Minocycline	-	20.5	53.9	32.9	47.2	-	-	-

Each datum point is an average from 1–6 experiments.

## Data Availability

Not applicable.
